# Multicenter prospective clinical study to evaluate children short-term neurodevelopmental outcome in congenital heart disease (children NEURO-HEART): study protocol

**DOI:** 10.1186/s12887-019-1689-y

**Published:** 2019-09-10

**Authors:** I. Ribera, A. Ruiz, O. Sánchez, E. Eixarch, E. Antolín, E. Gómez-Montes, M. Pérez-Cruz, M. Cruz-Lemini, M. Sanz-Cortés, S. Arévalo, Q. Ferrer, E. Vázquez, L. Vega, P. Dolader, A. Montoliu, H. Boix, R. V. Simões, N. Masoller, J. Sánchez-de-Toledo, M. Comas, J. M. Bartha, A. Galindo, J.M. Martínez, L. Gómez-Roig, F. Crispi, O. Gómez, E. Carreras, L. Cabero, E. Gratacós, E. Llurba

**Affiliations:** 1 Department of Obstetrics, Vall d’Hebron University Hospital, Universitat Autònoma De Barcelona, Barcelona, Spain; 2 Spain Maternal and Child Health Development Network, RETICS funded by the PN I+D+I 2013-2016 (Spain), ISCIII- Sub-Directorate General for Research Assessment and Promotion and the European Regional Development Fund (ERDF), ref. RD16/0022, Madrid, Spain; 3 BCNatal, Hospital Clínic of Barcelona and Hospital Sant Joan de Déu, Barcelona, Spain; 4 Division of Maternal and Fetal Medicine, Department of Obstetrics and Gynaecology, Hospital Universitario La Paz, Madrid, Spain; 5 Hospital Universitario 12 de Octubre, Universidad Computense de Madrid, Madrid, Spain; 6 Department of Paediatric Cardiology, Vall d’Hebron University Hospital, Universitat Autònoma De Barcelona, Barcelona, Spain; 7 Department of Pediatric Radiology, |Vall d’Hebron University Hospital, Universitat Autònoma De Barcelona, Barcelona, Spain; 8 Department of Pediatrics, Vall d’Hebron University Hospital, Universitat Autònoma De Barcelona, Barcelona, Spain; 9 Department of Neuropsicology, Vall d’Hebron University Hospital Barcelona, Barcelona, Spain; 10 Department of Cardiology, Hospital Sant Joan de Déu, Barcelona, Barcelona, Spain; 11 Universitary Hospital Germans Trias i Pujol, Barcelona, Spain; 12 Director of Obstetrics and Gynaecology Department, St Creu and St Pau Hospital, Sant Antoni Mª Claret, 167, 08025 Barcelona, Spain

**Keywords:** Congenital heart disease, Neurodevelopment, Predictive markers, Cardiac function and fetal brain MR

## Abstract

**Background:**

Congenital heart disease (CHD) is the most prevalent congenital malformation affecting 1 in 100 newborns. While advances in early diagnosis and postnatal management have increased survival in CHD children, worrying long-term outcomes, particularly neurodevelopmental disability, have emerged as a key prognostic factor in the counseling of these pregnancies.

**Methods:**

Eligible participants are women presenting at 20 to < 37 weeks of gestation carrying a fetus with CHD. Maternal/neonatal recordings are performed at regular intervals, from the fetal period to 24 months of age, and include: placental and fetal hemodynamics, fetal brain magnetic resonance imaging (MRI), functional echocardiography, cerebral oxymetry, electroencephalography and serum neurological and cardiac biomarkers. Neurodevelopmental assessment is planned at 12 months of age using the ages and stages questionnaire (ASQ) and at 24 months of age with the Bayley-III test. Target recruitment is at least 150 cases classified in three groups according to three main severe CHD groups: transposition of great arteries (TGA), Tetralogy of Fallot (TOF) and Left Ventricular Outflow Tract Obstruction (LVOTO).

**Discussion:**

The results of NEURO-HEART study will provide the most comprehensive knowledge until date of children’s neurologic prognosis in CHD and will have the potential for developing future clinical decisive tools and improving preventive strategies in CHD.

**Trial registration:**

NCT02996630, on 4th December 2016 (retrospectively registered).

## Background

Among congenital anomalies, congenital heart disease (CHD) is the leading cause of childhood mortality and morbidity, affecting up to 1% of all live births [1]. More than 36,000 infants with CHD are born in Europe every year and approximately 3000 more die in utero either spontaneously through legal abortion or during neonatal life [2]. Prognosis in these patients has significantly improved in recent years, with survival up to 95%, thanks to advances in diagnostic techniques and surgical management [3, 4].

Up to 50% of children with CHD present deficits in at least one area of neurodevelopment (learning ability, motor skills, language, etc.) [5, 6]. These findings seem to be more pronounced in CHD types associated with reduced oxygenated blood brain delivery, such as transposition of great arteries and left outflow tract obstruction; however, poor neurodevelopment has also been reported in children with heart defects with normal cerebral oxygen delivery [6].

Most studies evaluating neurological abnormalities in children with CHD have focused on factors associated with surgical repair [7], as abnormal neurodevelopment was long believed to occur as a result of the procedures associated with open surgery, mainly extra-corporeal circulation. However, signs of brain injury on magnetic resonance imaging and ultrasound have also been shown in prenatal studies [8, 9]. Additional studies report that CHD fetuses have smaller head biometries and signs of brain sparing from the second trimester of pregnancy, regardless of the type of CHD, supporting the recent hypothesis of early onset appearance of noxa mechanisms that could lead to a poorer neurodevelopment later in life in these children [10, 11].

Moreover, pregnancies affected with a CHD fetus have a higher rate of placental-related complications such as preeclampsia and preterm birth [12], which increases the chance of a poorer prognosis after birth and also after surgery [13]. Some authors have described an existing difference in plasma biomarkers in CHD, with higher ratio of anti-angiogenic factors compared to pro-angiogenesis factors and also increased blood levels of brain and heart hypoxemic biomarkers [14], leading to a possible related pathway between placental complications and CHD, without being clearly defined.

Despite the importance and frequency of these complications, the prenatal and postnatal risk factors impacting on neurodevelopment remain poorly understood. Studies from a clinical viewpoint have been based on small series. Consequently, research into whether differences exist in the spectrum of neurological damage according to type of cardiac defect and what kind of neurodevelopmental deficits these children present is needed.

Our hypothesis is that the results of NEURO-HEART study will provide the most comprehensive knowledge until date of children’s neurologic prognosis in CHD and will have the potential for developing future clinical decisive tools and improving preventive strategies in CHD to be used by cardiologists and obstetricians for parental assessment.

## Objectives

### Aims

The NEURO-HEART study aims to: 1) Describe the neuro-developmental outcome of patients with complex CHD at 24 months of age and identify a subgroup with poorer outcome. 2) Evaluate the utility of fetal and postnatal (preoperative and postoperative) diagnostic techniques for early recognition of patients at risk for suboptimal neurologic outcome.

## Methods

### Participants/eligibility criteria

The participation of different referral centers will permit: 1. sufficient recruitment of fetuses to be distributed into 3 groups depending on their CHD and prospective follow-up and integration of data from the prenatal period to early childhood.

The study protocol was approved by each of the Ethic Committee’s Centers, and written consent was obtained from all women to participate in the study.

### Settings/locations

- Hospital Universitari Vall d’Hebrón de Barcelona, Spain
- Hospital Universitario La Paz de Madrid, Spain
- Hospital 12 de Octubre de Madrid, Spain
- Hospital Maternitat-Clínic de Barcelona, Spain
- Hospital Sant Joan de Déu de Barcelona, Spain

### Recruitment and collection of data

Target recruitment is 150 cases classified in three groups according to the specific type of CHD. After reviewing existent literature and a variety of cohort classifications [15–17] and being aware of the capacity of recruitment due to our multicentric project, we decided to analyze patients with very similar diagnosis as it appeared inaccurate to create subgroups including different CHD types and will also make future parental assessment easier. Groups were made with our three most common diagnosis: TGV, TOF and LVOTO. These groups are known for having different patterns of blood supply to the brain, being TGA and hypoplasic left heart syndrome (HLHS) the ones with a higher oxygen impairment [15], so we expect to obtain differences depending on the CHD type. Cases are summarized in Table 1 in Appendix.

- Group 1: Transposition of great arteries.
- Group 2: Tetrallogy of Fallot.
- Group 3: Left outflow tract obstruction.

A control group of 50 low-risk pregnancies will also be recruited. These patients will be followed up once a month with fetal ultrasound at each visit; functional echocardiography and MRI, performed at the same times as in the case group.

See Table 1 in Appendix. Classification according to CHD type.

#### Exclusion criteria

Pregnancies with gestational age < 20 weeks or > 38 weeks, associated non-cardiac malformations, presence of chromosomal abnormalities, associated arrhythmia and maternal conditions that might affect fetal hemodynamics such as diabetes, thyroid disease or preeclampsia, multiple pregnancy and fetal anemia. Minimum age of inclusion for the study will be 18 years old.

#### Database

The local research coordinator and/or the staff from participating hospitals will identify eligible women and after counseling and reading the information form, patients will be asked for written consent.

At study recruitment, demographic, obstetric and medical history data will be recorded into a web-based Case Report Form (CRF) that will be accessible through a restricted website. Details on delivery, maternal and neonatal assessments during pregnancy or post-partum data will be recorded in the CRF. The collected data will be coded and processed with adequate precaution to ensure patient confidentiality with the following measures:

Initials of participants as well as a local patient number will be recorded in the electronic database. Linking names with patients’ numbers will only be available in the local clinics. Each participating clinic will receive a login name and password to gain access to the web-secured database. Database access will be restricted to clinicians with electronic password. Full access to the entire database will also be restricted to some members of the research staff.

### Procedures and interventions

#### Data collection

Patients will be followed up at the obstetrics and fetal cardiology unit every 4 weeks with a multidisciplinary approach. At inclusion, a blood sample from both parents will be taken. During pregnancy, visits will be scheduled every 4 weeks, including fetal biometries and Doppler. At 28–32 weeks of pregnancy, a functional echocardiography will be performed and finally, at 35–37 weeks, a MRI for evaluating fetal brain will be done.

At delivery, a cord blood and a maternal blood sample will be taken.

Mode of delivery, perinatal complications, biometric data such birth weight and head circumference, need for intubation or need for drugs after birth will also be recorded. A FCUS will be performed during the ICU stay. Blood samples will be taken and continuous EEG for 2 h will be performed before and after surgery. Brain oximetry will be measured from 12 h before to 48 h after surgery.

Children’s follow-up will take place at 12 and 24 months of corrected age. On both appointments, electroencephalogram (EEG) and functional echocardiography will be performed and a blood sample will be obtained. At 2 years of age, a MRI will be performed.

Neurodevelopment follow-up will be performed at 12 months of age using the Ages & Stages Questionnaires (ASQ) and at 24 months of age using Bayley-III Scale, applied by a previously trained neuropsychologist. These scores are widely used to determine the child’s performance compared with norms taken from typically developing children of the same age (in months).

**Fig. 1:**
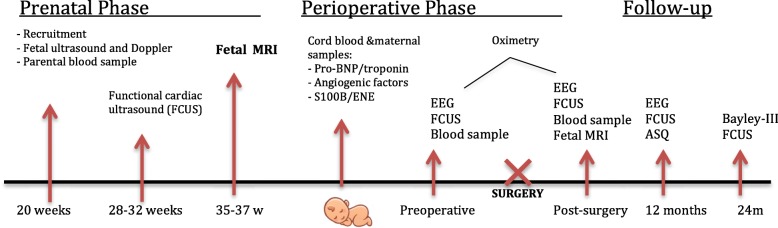
Study procedures flowchart

Study procedures will be performed and organized as shown in Fig. 1.

#### Ultrasound evaluation

Gestational age at scan will be calculated based on the crown-rump length obtained at first trimester screening [18]. A complete ultrasound examination will be performed in each fetus using high quality ultrasound systems.

Fetal biometric parameters assessed will include: biparietal diameter (BPD), head circumference (HC), abdominal circumference (AC) and femoral length (FL). Estimated fetal weight will be calculated according to the method of Hadlock et al. [19]; both estimated fetal weight and birth weight centile will be obtained using local reference curves [20]. Doppler recordings will be made in the absence of fetal movements with voluntarily suspended maternal breathing. Measurements of pulsed Doppler parameters will be taken automatically from three or more consecutive waveforms, with an angle of isonation as close to 0° as possible. Umbilical artery (UA) will be evaluated in a free loop of the umbilical cord; middle cerebral artery (MCA) will be measured in a transverse view of the fetal skull at the level of its origin from the circle of Willis [21]. Cerebroplacental ratio (CPR) will be calculated by dividing MCA by UA pulsatility index (PI) [22]. Uterine artery (UtA) will be evaluated with the probe placed on the lower quadrant of the abdomen, angled medially, with identification by color Doppler imaging of the apparent crossover with the external iliac artery. Mean uterine artery PI will be calculated as the average PI of right and left arteries [23].

Fetal echocardiographic examination will be performed according to the International Society of Ultrasound in Obstetrics and Gynecology guideline [24]. Briefly, cardiac axis and situs, pericardial effusions, ventricular morphology, veno-atrial, atrioventricular and ventricule-arterial connections, size and relationships of left and right ventricular outflow tracts, ductal and aortic arches, atrial and interventricular septum and flow through atrioventricular and semilunar valves will be evaluated using two-dimensional, color and pulsed Doppler ultrasound. Prenatal diagnosis of CHD will be confirmed using postnatal echocardiography or autopsy in cases of neonatal death.

Functional echocardiographic scan will include several measures [25]: from a transverse 4-chamber view in M-mode: end-diastolic diameter (EDD), end-systolic diameter (ESD), septum, myocardial wall thickness in systole and diastole (calculation of shortening (SF) and ejection fractions (EF)). From an apical/basal 4-chamber view in 2D: cardiothoracic ratio, atrial areas, AV valve diameters, ventricular longitudinal and transverse diameters (calculation of sphericity indices). With an apical/basal 4-chamber view, using pulsed Doppler, we will measure early (E) and late (A) peaks of transvalvular filling velocities of left and right ventricles (E/A ratio calculation) and the presence of mitral/tricuspid insufficiency and its velocity. From an apical/basal 4-chamber view in M-mode: mitral and tricuspid annular-plane systolic excursion (MAPSE, TAPSE) will be measured. With an apical/basal 5-chamber view in 2D, aortic valve diameter in systole will be measured. Finally, with an apical/basal 5-chamber view adding pulsed Doppler, we will measure aortic peak systolic velocity, measurement of the velocity time integral (VTI), left fetal heart rate (calculation of left cardiac output (CO)), left isovolumetric contraction (ICT) and relaxation (IRT) times and ejection time (ET) (calculation of myocardial performance index (MPI)). Pulmonary valve diameter in systole, pulmonary artery peak, systolic velocity, VTI and right fetal heart rate (calculation of right CO, combined cardiac output (CCO) and cardiac index (CI)) will also be measured. With a 3-vessel and trachea view in 2D, the aortic isthmus and ductus arteriosus diameters will be measured. In the same plane with pulsed Doppler, aortic isthmus PI, systolic and diastolic VTI (calculation of isthmus flow index (IFI)) and PI index will be recorded.

Myocardial peak systolic (S′) and diastolic (E’, A’) velocities and myocardial performance index (MPI’) in mitral, septal and tricuspid annuli will be also obtained by tissue Doppler imaging with described previous methodology [26].

#### MRI evaluation

Pregnant women will undergo fetal MRI on 1.5 Tesla scanners at 35 to 37 weeks of gestational age following the American College of Radiology guidelines for the use of medical imaging during pregnancy and lactation [27].

T2-weighted images (HASTE) will be acquired in sagittal, axial and coronal sections including all the brain at 3.5 mm of thickness, as reported previously [28]. MR spectroscopy (MRS) images will also be acquired in the frontal lobe. Anatomical 2D images in sagittal, axial and coronal sections will be acquired for volumetric reconstruction. An anatomical control sequence (HASTE) will be taken to verify the position of the fetal head has not significantly changed, in which case the MRS images will have to be repeated. Diffusion and MRS data will additionally be acquired if possible. If the quality of the images is suboptimal, sequences will be repeated.

Structural MRI images will be reviewed for the presence of anatomic abnormalities by an experienced neuroradiologist. Parameters evaluated will be: brain calcifications if present, hypoxic or ischemic damage. Brain biometric measurements will be performed using the semiautomatic Analyze 9.0 software (Biomedical Imaging Resource; Mayo Clinic, Kansas City, KS) by an experienced examiner blinded to diagnosis. Cortical fissure delineations in the fetal MRI will be performed adapting previously described methodology to assess cortical development in fetal ultrasound [29, 30] and will include the following measures, performed as they have previously been described for our group [31]: BPD, fronto-occipital diameter (FOD), occipital fossa, cerebellum diameter and lateral ventricular size, Sylvian fissure depth, parieto-occipital suture depth and insula measurements. In a middsagital plane, corpus callosum longitudinal measurement and vermix diameter biometries will be performed. Calcarine and Cingular fissures depth will be measured in coronal plane. All biometric parameters will be corrected by BPD and therefore expressed as ratios. Cortical fissure depths will be measured bilaterally.

MRS data will be acquired from the semi oval centre, with single voxel PRESS localization and will be processed in 2 ways using MATLAB R2010a (MathWorks Inc., Natick, MA): method A, standard averaging of 128 transients (first free-induction-decay block discarded); and method B, spectral sorting (discarding data with gross artifacts and lipid contaminations) and alignment (based on the residual water peak), before averaging. Spectra will then be analyzed for quality assessment and selection: both with jMRUI v4.0 (MRUI Consortium, EU), spectral pattern well resolved for major peaks, such as choline compounds, total creatine, and N-acetylaspartate, and amplitude ratio of lipid (1.3–0.9 ppm) to choline peak (3.21 ppm) < 10%; and with LCModel v6.3 (Stephen Provencher Inc., Oakville, Canada), peak full-width at half-maximum < 0.1 ppm and signal-to-noise ratio [32].

Postnatal MRI will be performed with a Siemens Magnetom Trio 3.0 Tesla (Erlangen, Germany). T1 sequences will be taken for anatomical data. Qualitative analysis on T2 and FLAIR frequencies will be made with FSL software (functional magnetic resonance imaging of the brain’s Software Library) [33]. MPRAGE imaging will be used for grey matter quantitative measurements and DTI images for white matter measurements.

The presence of myelinisation in conventional sequences, brain cortical development, ischemic or hemorrhagic lesions and total grey /white matter volume will be recorded. In addition, metabolic analysis will be made and compared to data published in previous studies [34]. All volumetric estimations will be obtained using Cavalieri’s principle 27 by a multiplanar analysis considering a slice thickness of 3.5 mm with no gap interval between them. For brain maturation evaluation, specific MRI visual scores will be used [35].

#### Plasma biomarkers analysis

At the time of inclusion, a maternal venous blood sample will be drawn. Additionally, a second blood sample will be taken from the mother at the time of delivery. Blood samples from children with CHD will be drawn at different time points: 1. after birth (cord blood) 2. during the pre-surgery period and 3. immediately after surgery. All blood samples will be processed within 1 h. Plasma will be separated by centrifuge at 1400 g for 10 min and stored at 4 °C.

Angiogenic Placental Growth Factor (PlGF) and antiangiogenic soluble fms-like tyrosine kinase-1 and soluble endothelin (sFlt1, sEng) factors will be measured in pregnant patients from both case and control groups. In cord blood, the following biochemical markers will be measured: a. markers of cardiac function and injury: Troponin I, N-Terminal pro-brain natriuretic peptide (NT-proBNP), endothelin-1 and heart fatty acid-binding protein (h-FABP); b. neurological damage markers S-100 proteins and neuron-especific enolase (ENE) and c. angiogenic PlGF and antiangiogenic sFlt1 factors.

All plasma biomarkers will be determined by ELISA commercial kits following the manufacturer’s instructions. Angiogenic factors (PlGF, sFlt-1 and sEng) are obtained from R&amp; D Systems (R&amp;D Systems Europe Ltd., Abington, UK). Markers of cardiac function and injury are obtained from different manufacturers: Troponin I from AbFRONTIER (Young In Frontier Co. Ltd. Seoul, Korea), NT-proBNP from BG (BlueGene biotech CO. Ltd., Shanghai, China), Endothelin-1 from R&amp;D Systems (R&amp;D Systems Europe Ltd, Abington, UK) and h-FABP from Hycult Biotech (Hycult Biotech, Uden, The Netherlands). Markers neurological damage (S100B and NSE) are obtained from BG (BlueGene biotech CO. Ltd., Shanghai, China).

#### Neonatal evaluation and neurodevelopment

During hospital stage, data on intensive care unit and hospital length stay, need for mechanic ventilation, need for vasoactive drugs and mortality will be recorded.

Surgical duration, time on extracorporeal support, cardiac ischemia time and the need and duration of circulatory arrest will be also recorded. Need for renal replacement therapies, extracorporeal life support and cardiac arrest before or after surgery will be also recorded.

Monitoring for oximetry and EEG will be recorded before and after surgery. Neurosonography will be used to evaluate brain anatomy and findings such as: ventriculomegaly (VMG) and / or increased subarachnoid space; signs of periventricular leukomalacia (PVL) signs of intraventricular hemorrhage (IVH); presence of calcifications and alterations in cortical development.

A baseline EEG of 12 channels will be performed preoperatively and within the first 48 h postoperatively. Presence and number of power crisis and overall duration of delta waves will be recorded.

Following previous literature by Mebius et al. [36], regional cerebral oximetry using technology Near-infrared Spectroscopy (NIRSS) will be recorded continuously. NIRSS has been described as a useful predictor for cardiac arrest in patients with complex CHD previous to surgery and a reliable way to measure brain oxygen saturation. Bilateral forehead sensors will be placed preoperatively (2 h prior to surgery) and will be kept for at least 24 h post-operatively. Global percentage of time below a threshold of 45 and percentage of time below 20% of the baseline measurement will be recorded.

Children will be followed up for 2 years. Cognitive neurodevelopment will be assessed at 1 year of age using the ASQ. This test is validated and extensively used as a screening method for detecting impaired neurodevelopment in high-risk children. Research nurses will be notified when a patient will approach the corrected age of 1 year by an email generated from our database. After this alert, children’s parents will be called to complete again the questionnaire. In case the parents do not return the questionnaire, a reminder will be sent. We will be using the validated Spanish translation of the ASQ, covering the age-range 4–60 months.

In the ASQ 3rd edition surveys, parents are asked to answer “YES” (10 points), “SOMETIMES” (5 points) or “NOT YET” (0 points) to a series of items of the following categories of global neurodevelopment: communication, large movements, fine movements, problem solving and socio-individual competence. A total score is obtained from the sum of the items of each category. There is a different baseline for every age and category under which an alteration in this part of the neurodevelopment is considered to exist. In our study, results were analyzed as: a) impairment in each category; or b) impairment of at least one category. Children scoring under 2 standard deviations (SD) will be referred to diagnostic assessment.

At 2 years of age, Bayley-III Scale will be performed by a previously trained neuropsychologist. This measure consists on different developmental play tasks administered between 45 to 60 min and derives in a developmental quotient (DQ) rather than an intelligence quotient. Raw scores of successfully completed items are converted to scale scores and to composite scores. These scores are used to determine the child’s performance compared with norms taken from typically developing children of their age (in months). The most recent edition, the Bayley-III, has three main subtests: 1. the Cognitive Scale, which includes items such as attention to familiar and unfamiliar objects, looking for a fallen object, and pretend play, 2. the Language Scale, which taps understanding and expression of language and 3. the Motor Scale, which assesses gross and fine motor skills. The scaled score ranges from 1 to 19 points, with a mean of 10 points in the validated population (USA), and a SD of 3 points. Some studies question the capacity of the Bayley Scale, especially the 3rd edition, for detecting impaired neurodevelopment due to a tendency to undervalue alterations [37]. For this reason, our study defined impaired cognitive neurodevelopment as a score of 8 or less on the scale (25th percentile, equivalent to the mean minus 0.66 SD).

FCUS and MRI will also be performed at 2 years of age.

At the same period of time, low-risk pregnant women attending a routinely 20-weeks scan at Vall d’Hebron Obstetrics Department would be asked for participation as part of the control group of the study. In this group of women, a blood sample at the time of the inclusion and during delivery, as well as cord blood sample, will be obtained. During pregnancy, visits will be scheduled every 4 weeks and will include fetal biometries and Doppler measurements. At 28–32 weeks of pregnancy, a functional echocardiography will be performed and finally, at 35–37 weeks, a fetal brain MRI will be done. No further maternal or neonatal tests will be performed.

## Outcome measures

### Primary outcome mesures

A. Neurodevelopmental outcome according to CHD types.

1. Abnormal ASQ: ASQ score results will be compared to the normal Bell curve results for the same age. Those close to the cut-off of 2 SD will be considered at risk for neurodevelopment impairment.
2. Bayley-III test will be considered at risk for neurodevelopment delay when scored 8 or less on the scale (25th percentile, equivalent to the mean minus 0.66 SD)

### Secondary outcome mesures

1. Hemodynamic changes and Doppler fetal cardiac function in fetuses with CHD by type.
2. Cortical brain development assesed by MRI such as changes in fissure depths, head biometries or brain metabolism spectroscopic parameters.
3. Fetal head biometries and perfusional parameters.
4. Biochemical markers in cord blood associated with neurological damage in children with CHD.
5. Parameters of perioperative brain.
6. Severe neonatal or child morbidity will be taken into consideration as primary outcome in our trial. The presence of one or several of the following complications will define severe morbidity: severe respiratory distress, intraventricular hemorrhage grades III-IV, treated ductus arteriosus persistence, renal dysfunction, necrotizing enterocolitis, intestinal perforation, early and late sepsis, retinopathy of prematurity treated with laser, bronchopulmonary dysplasia, periventricular leucomalacia, postnatal administration of corticosteroids or inotropic drugs and/or death.
7. Statistical association of all the parameters described in 1–5 with neurological development.
8. Regression statistical measures to select the most valuable variables related to neurological impairment in order to identify risk factors for brain damage in a preoperative stage.

All these measures will be analyzed in an exploratory manner. Analysis will be performed to investigate the utility of pre- and post-natal markers in the short-term prediction of adverse neurological outcomes in order to create prognostic tools for early detection of patients at risk.

### Statistical analysis

#### Sample size

All participating centers together have over 25,000 deliveries / year and more than 150 births of congenital heart defects babies We have planned a 3 year-period recruitment that, considering our previous birth statistics, cases of interruption of pregnancy and an estimated 10% loss of follow up, will allow us to recruit 60 patients diagnosed with TGA, 30 patients with TOF and 30 patients for the LVOTO group, the largest series published so far.

#### Data analysis

Storage of variables will be done in a database specially designed for our study by the Department of Bioinfomatics and Clinical Pharmacology (HUVH). Once the sample collection and conducting of laboratory determinations will be finished, we will proceed to the evaluation and analysis of the results obtained with help and advice by Bioinformatics Research Institute Unit and the Support for Investigation Unit (USMI) from Vall d’Hebron Hospital.

#### Statistical analysis

Data will be collected, digitalized and stored in a data base designed for the study. The statistical analysis will be based on single variable descriptive models. In case of finding associations with confusion factors, multivariable models will be considered.

All statistical comparative analysis will be done using SPSS 2.13 (SPSS for Windows, SPSS Inc., Chicago, Ill., USA) and MatLab statistical programs (2007b, The MathWorks Inc., Natick, USA). In hypothesis testing for population inference, statistical significance of 0.05 will be postulated.

For the analysis of blood sample biomarkers all parameters will be transformed to MoMs as well as ultrasound biometric and Doppler parameters will be converted to z-scores using previously published data [38–40] in order to compare data obtained in different gestational age. MRI measurements will be corrected by BPD and birth weight centile.

For multiple comparsions between groups one-way ANOVA tests will be performed. In case of non-parametric data, Mann-Whitney tests will be used.

Also multivariate analysis using logistic regression will be made with the support of the Unidad de Soporte en Metodología para la Investigación Biomédica (USMIB) using the application STATA v.11.Different analyses will be carried out in order to establish the association and prediction power of the characteristics analyzed in all tests independently and also combined by implementing classical statistic methods (linear and logistic regression) and inference methods with and without a priori assumptions (Bayesian networks, decision trees, principal component analysis among others). After that, the results will be implemented in a learning machine for algorithms in MATLAB (2007b, The MathWorks Inc., Natick, USA). The type of analysis will depend on each specific objective.

#### Limitations of the study

Establishing specific associations to a single CHD type may require a larger sample size than we will be able to recruit, especially in the Fallot and LVOTO groups. Although our time to include patients may not be enough to obtain the necessary number of cases, we believe they will still be remarkable series and a unique opportunity to create a CHD population cohort.

We are aware that the LVOTO group is intrinsically heterogenical with different prognosis and probably also different brain development impairment between a coartation of aorta and a hypoplasic heart syndrome case, but this pathophysiological classification allows a reasonable approach.

Inclusion of healthy controls with the same follow up has its logical limitations, and due to ethical considerations, no control children would be used for biochemical markers.

## Discussion

Congenital heart disease is one of the leading causes of congenital malformation. While advances in early diagnosis and postnatal management have increased survival in CHD children [3], worrying long-term outcomes, particularly neurodevelopmental disability, have emerged as a key prognostic factor in the counseling of these pregnancies [41, 42].

A recent study showed that a high proportion of fetuses with CHD already have a smaller head and increased brain perfusion in the second trimester of pregnancy [15]. Additionally, a substantial percentage of newborns with CHD have signs of brain injury on magnetic resonance imaging and reduced cranial size [43], suggesting an early onset of the mechanisms leading to poorer neurodevelopment later in life [44]. It has been hypothesized that altered cerebral perfusion is one of the main contributors to abnormal neurodevelopment in fetuses with CHD. Although abnormal head size was more pronounced in fetuses with compromised blood delivery to the brain, it was also present in milder forms of CHD, suggesting that there could be additional mechanisms that contribute to abnormal neurodevelopment in CHD cases [12, 45–47].

No prospective studies have been performed to allow clinicians to do a good assessment on neurological prognosis with compelling evidence until now. Differences in brain development markers have not been reported in previous series, but we believe those results might be related to sample size [48].

The Neuro-Heart trial aims to compare and describe preoperative markers on CHD affected fetuses both prenatal as well as postnatal brain functional monitoring during the perioperative period and through cardiac surgery. At the same time, this multicentric trial will allow CHD patients included to be classified into specific CHD groups in the largest series published so far.

## Data Availability

The datasets generated during and/or analyzed during the current study are not publicly available due to patient confidentiality, but are available from the corresponding author on reasonable request.

## Appendix

**Table 1:** Classification according to CHD type

Group 1: Transposition of great arteries	
• With or without VSD.	
Group 2: Tetralogy of Fallot	
Group 3: LVOTO	
• Coartation of aorta	
• Aortic arch hypoplasia	
• Double-inlet left ventricle with coartation/aortic arch hypoplasia	
• Double outlet right ventricle with coartation/aortic arch hypoplasia	

## Abbreviations

AC: Abdominal circumference
ASQ: Ages and stages questionnaire
AVSD: Atrioventricular septal defect
BPD: Biparietal diameter
CCO: Combined cardiac output
CHD: Congenital Heart Disease
CI: Cardiac index
CO: Cardiac output
CPR: Cerebroplacental ratio
DQ: Developmental quotient
EDD: End-diastolic diameter
EEG: Electroencephalogram
EF: Ejection fraction
ENE: Neuron-especific enolase
ESD: End-systolic diameter
ET: Ejection time
FCUS: Functional cardiac ultrasonography
FL: Femoral length
FOD: Fronto-occipital diameter
HC: Head circumference
h-FABP: Heart fatty acid-binding protein
HLHS: Hypoplasic Left Heart Syndrome
ICT: Isovolumetric contraction time
IFI: Isthmus flow index
IQ: Intelligence Quotient
IRT: Isovolumetric relaxation time
IVH: Intraventricular hemorrhage
LVOTO: Left Ventricular Outflow Tract Obstruction
m: Months
MCA: Middle cerebral artery
MPI: Myocardial performance index
MRI: Magnetic resonance imaging
NT-proBNP: N-Terminal pro-brain natriuretic peptide
PI: Pulsatility index
PlGF: Angiogenic placental growth factor
PVL: Periventricular leukomalacia
SD: Standard deviations
sEng: Soluble endothelin
SF: Shortening fraction
sFlt: Antiangiogenic soluble fms-like tyrosine kinase-1
TGA: Transposition of the great arteries
TOF: Tetralogy of Fallot
UA: Umbilical artery
UtA: Uterine artery
VMG: Ventriculomegaly
VSD: Interventricular communication
VTI: Velocity time integral
W: Weeks
